# Asynchrony Between Endometrial miRNA- and mRNA-Based Receptivity Stages Associated with Impaired Receptivity in Recurrent Implantation Failure

**DOI:** 10.3390/ijms26157349

**Published:** 2025-07-30

**Authors:** Yu-Jen Lee, Chi-Ying Lee, En-Hui Cheng, Wei-Ming Chen, Pok Eric Yang, Chun-I Lee, Tsung-Hsien Lee, Maw-Sheng Lee

**Affiliations:** 1 Genetic Diagnosis Laboratory, Lee Women’s Hospital, Taichung 40652, Taiwan; yujen1027@ivftaiwan.com (Y.-J.L.); s110080811@m110.nthu.edu.tw (C.-Y.L.); enhuicheng@gmail.com (E.-H.C.); 2 Department of Post-Baccalaureate Medicine, National Chung Hsing University, Taichung 40227, Taiwan; 3 Institute of Bioinformatics and Structural Biology, National Tsing Hua University, Hsinchu 30013, Taiwan; 4 Inti Labs, Hsinchu 30261, Taiwan; wmchen@intilabs.com (W.-M.C.); eric@intilabs.com (P.E.Y.); 5 Department of Obstetrics and Gynecology, Chung Shan Medical University Hospital, Taichung 40201, Taiwan; cshy1569@csh.org.tw (C.-I.L.); msleephd@csmu.edu.tw (M.-S.L.); 6 Division of Infertility, Lee Women’s Hospital, Taichung 40402, Taiwan; 7 School of Medicine, Chung Shan Medical University, Taichung 40201, Taiwan; 8 Institute of Medicine, Chung Shan Medical University, Taichung 40201, Taiwan

**Keywords:** endometrial receptivity, microRNA, messenger RNA, recurrent implantation failure, embryo transfer timing

## Abstract

Understanding the molecular basis of endometrial receptivity is crucial for improving implantation outcomes in assisted reproduction, especially for patients with recurrent implantation failure (RIF). This study investigates the timing relationship between microRNA (miRNA) and messenger RNA (mRNA) profiles in the endometrium using simultaneously the endometrial receptivity array (ERA) and the microRNA receptivity assay (MIRA) in 100 RIF patients undergoing euploid blastocyst transfer. The concordance rate between ERA and MIRA was 72% (Kappa = 0.50), suggesting partial overlap in profiling. Patients were stratified by the timing sequence of miRNA relative to mRNA into Fast, Equal, and Slow groups. Those with delayed miRNA expression (Slow group) had significantly lower pregnancy rates (54.5%) than those with synchronous or leading miRNA expression (81.9% and 94.1%, respectively; *p* = 0.031). Moreover, the Slow group exhibited higher prior implantation failure counts and altered expression in 15 miRNAs, many involved in aging-related pathways. These findings highlight that asynchronous miRNA–mRNA profiles may reflect impaired receptivity and suggest that miRNA-based staging adds valuable diagnostic insight beyond mRNA profiling alone. Dual assessment of mRNA and miRNA profiles may offer additional diagnostic insight into endometrial receptivity but requires further validation before clinical application.

## 1. Introduction

Embryo implantation is a critical step in the process of achieving a successful pregnancy, and its success is largely dependent on the receptivity of the endometrium [1,2,3]. Endometrial receptivity refers to the window of implantation (WOI), a limited time frame during which the endometrium is optimally prepared to allow the embryo to attach and implant. This period is regulated by a complex interplay of hormonal signals and gene expression changes within the endometrial tissue [4]. For many years, the focus of research on endometrial receptivity has been centered around the expression of messenger RNA (mRNA) profiles, which are considered the primary drivers of protein synthesis and cellular function [5]. However, the role of microRNAs (miRNAs), which are small non-coding RNAs involved in the post-transcriptional regulation of gene expression, has recently garnered attention for their potential involvement in endometrial receptivity [1,6].

The traditional approach to assessing endometrial receptivity involves analyzing mRNA expression patterns using tools such as the endometrial receptivity array (ERA) [7]. The ERA evaluates the expression levels of specific genes known to be associated with the receptive state of the endometrium. This method has been adopted in clinical practice to provide a personalized window for embryo transfer (ET) in in vitro fertilization (IVF) cycles. Nonetheless, recent ESHRE guidelines and HFEA ratings caution against its routine use due to limited evidence supporting improved outcomes [8]. In such conditions, the true meanings of ERA findings and the regulatory mechanism deserve further investigation. Since mRNA stability and translation, together with the subsequent protein expression and cellular function, are affected by the regulatory mechanisms exerted by miRNAs [9], the expression profiles of miRNA in the endometrium are valuable research targets.

MiRNAs have emerged as key regulators of gene expression in various biological processes, including cell proliferation, differentiation, and apoptosis [10]. In the context of endometrial receptivity, miRNAs may modulate the expression of genes involved in preparing the endometrium for implantation [9,11]. The intricate balance between miRNA and mRNA expression is crucial for maintaining the proper function of the endometrial tissue during the WOI. Disruptions in this balance, particularly in the timing of miRNA and mRNA expression, could lead to a suboptimal receptive state, contributing to implantation failure [11,12].

Extensive research has been conducted on the role of mRNA in endometrial receptivity. Studies have identified specific mRNA expression profiles associated with the different phases of the WOI, leading to the development of diagnostic tools like the ERA [7]. These tools have significantly advanced the understanding and clinical management of endometrial receptivity, particularly in patients with recurrent implantation failure (RIF) [9]. The ERA, for instance, categorizes the endometrial environment into pre-receptive, receptive, and post-receptive phases based on the expression of a panel of genes. This categorization allows clinicians to optimize the timing of embryo transfer, thereby increasing the likelihood of successful implantation [13,14].

However, the role of miRNAs in endometrial receptivity has not been fully explored. Emerging evidence suggests that miRNAs play a significant role in regulating the gene expression networks that govern endometrial receptivity [9,11]. Several studies have identified differentially expressed miRNAs in the endometrium during the WOI, implicating their involvement in processes such as immune modulation, cell adhesion, and tissue remodeling—all of which are critical for successful implantation [11,15,16]. Despite these findings, there is a paucity of research on how the timing of miRNA expression relative to mRNA expression affects endometrial receptivity and pregnancy outcomes [9].

The interplay between miRNAs and mRNAs is complex and involves both direct and indirect regulatory mechanisms [10]. MiRNAs can bind to complementary sequences on target mRNAs, leading to mRNA degradation or inhibition of translation. This regulatory effect can fine-tune gene expression, ensuring that proteins are synthesized at the right time and in the right amount [10]. In the context of endometrial receptivity, the timing of miRNA and mRNA expression is particularly important [9,12]. A delay in miRNA expression relative to mRNA could result in the prolonged expression of proteins that should be downregulated as the endometrium transitions from a proliferative to a receptive state [11,15,17]. Conversely, premature miRNA expression could suppress the synthesis of proteins necessary for establishing a receptive endometrium [18,19].

The challenge of RIF in assisted reproductive technology (ART) remains a complex clinical phenomenon with significant implications for patients and clinicians. Despite decades of research, the absence of a universally accepted definition complicates both clinical management and research standardization. The most common quantitative criterion involves ≥3 consecutive failed IVF/ICSI cycles with high-quality embryo transfers (1–2 embryos per cycle) [20]. ESHRE’s 2023 guidelines emphasize individualized prognoses, suggesting RIF should be diagnosed when cumulative implantation probability falls below 60% after transfers adjusted for age and embryo quality [8]. Recent critiques argue that requiring ≥3 euploid blastocyst failures better isolates true implantation pathology, as aneuploidy accounts for 50–80% of losses in women >35 years [21,22]. Therefore, patients with RIF undergoing euploid blastocyst transfer (EBT) may represent an ideal population for studying endometrial receptivity.

In addition to the ERA using mRNA profiles to determine the endometrial receptivity stages, another miRNA-based tool, MIRA, was developed for the same purpose. Given the potential importance of miRNA–mRNA interplay in endometrial receptivity, it is essential to investigate the synchrony or concordance between these two endometrium receptivity staging systems. We hypothesize that discordance or asynchrony between miRNA- and mRNA-based receptivity staging may be negatively associated with pregnancy outcomes. Understanding how the asynchrony between miRNA and mRNA expression impacts pregnancy outcome could lead to the development of more sophisticated diagnostic tools for endometrium receptivity and personalized treatment strategies for patients with RIF undergoing EBT.

## 2. Results

### 2.1. Concordance Between mRNA (ERA) and miRNA (MIRA) Profiles

In this study, we compared the endometrial receptivity stages of patients with recurrent implantation failure (RIF) using two diagnostic methods: MIRA (89 microRNA profiles) and the ERA (238 mRNA profiles). We categorized patients into pre-receptive (P + 4), receptive (P + 5), and post-receptive (P + 6) groups based on their ERA or MIRA results. The distribution was as follows: 22% pre-receptive, 65% receptive, and 13% post-receptive by the ERA, and 20% pre-receptive, 64% receptive, and 16% post-receptive by the MIRA. Concordance between the two diagnostic tools was observed in 72% of the samples, with a Kappa statistic of 0.50 (95% CI: 0.34–0.66), indicating moderate agreement between these two methods (Table 1).

### 2.2. Staging Synchrony Between mRNA (ERA) and miRNA (MIRA) Profiles

To determine the staging synchrony between miRNA and mRNA, we grouped the patients into Fast [(P + n)mi vs. (P + j)m, n < j], Equal [(P + n)mi vs. (P + j)m, n = j], and Slow [(P + n)mi vs. (P + j)m, n > j] categories (n or j = 4 or 5 or 6). In other words, ‘Fast’ indicates miRNA-defined receptivity stage earlier than mRNA-defined stage, ‘Equal’ indicates concordant stages, and ‘Slow’ indicates delayed miRNA-defined stage relative to mRNA-defined stage. Notably, the Slow group, characterized by lagged miRNA profile expression relative to mRNA profiles, show a significantly lower clinical pregnancy rate following frozen embryo transfer (FET) compared to the Fast and Equal groups (54.5% vs. 94.1% and 81.9%, respectively; *p* = 0.031) (Table 2). The post hoc comparison of the clinical pregnancy rates between the Fast and Slow groups (16/17 vs. 6/11, *p* = 0.014 by X^2^ test) is still significant after Bonferroni correction (*p* values < 0.05/3).

### 2.3. Clinical Factors Associated with Lagging miRNA-Based Relative to mRNA-Based Receptivity Staging

We further examined the association of clinical factors with lagging miRNA profile expression relative to mRNA profiles (Table 3). The Slow group had fewer instances of endometrial polyps (2/11 = 18.2% vs. 23/72 = 31.9% vs. 10/17 = 58.8%, *p* = 0.019 by X^2^ for trend test).

### 2.4. Function Enrichment Analysis for Those miRNAs with Differential Expression in the Slow Group

**Figure 1:**
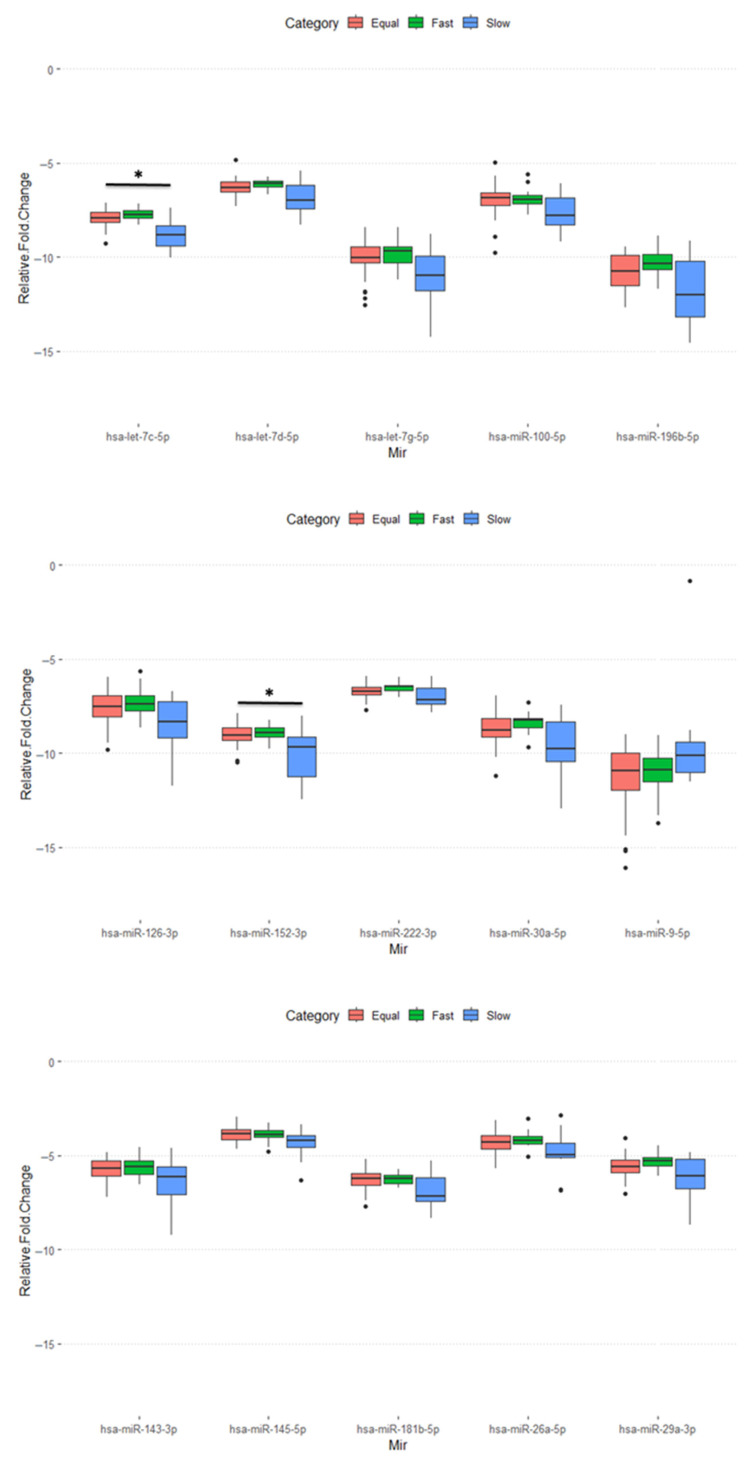
Expression analysis of the miRNA panel (total 89 miRNA) across three groups defined by the timing concordance between MIRA and ERA results for endometrial receptivity: the Fast group (MIRA indicates receptivity earlier than ERA), the Equal group (MIRA and ERA concordant), and the Slow group (MIRA indicates receptivity later than ERA). Eighteen miRNAs were found to be differentially expressed among the groups. Of these, fifteen miRNAs (miR-100-5p, miR-9-5p, miR-196b-5p, let-7c-5p, let-7d-5p, let-7g-5p, miR-126-3p, miR-222-3p, miR-152-3p, miR-145-5p, miR-143-3p, miR-181b-5p, miR-29a-3p, miR-30a-5p, and miR-26a-5p) exhibited significantly altered expression in the Slow group compared to both the Fast and Equal groups (*p* < 0.05). Notably, all of these miRNAs—except for miR-9-5p—were significantly downregulated in the Slow group. The * in the figure indicates *p* < 0.001.

**Figure 2:**
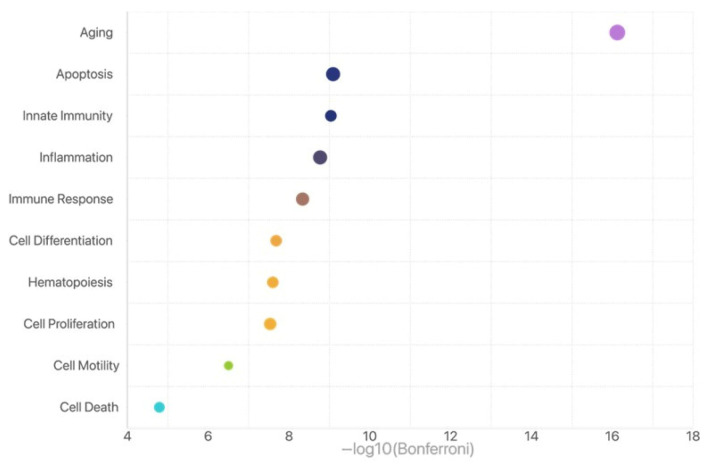
Gene Ontology (GO) enrichment analysis of differentially expressed miRNAs. The analysis reveals that “Aging” is the most statistically significant enriched biological process (*p*-value < 10^−15^), followed by other related processes such as Apoptosis, Innate Immunity, Inflammation, and Immune Response. These GO terms are biologically interconnected: Aging is an upstream physiological process that can drive changes in Apoptosis and Immune Response, and is closely associated with chronic low-grade Inflammation (also known as inflammaging). This network of processes may reflect age-related alterations in the endometrial immune microenvironment and cell regulatory mechanisms, which could contribute to the observed discordance between miRNA and mRNA expression profiles. The prominence of Aging in the enrichment results supports the hypothesis that miRNA-mediated regulatory changes associated with aging may underlie temporal shifts in endometrial receptivity (e.g., delayed receptivity in the MIRA–ERA Slow group). Bubble size represents the number of miRNAs associated with each GO term. The x-axis shows the negative logarithm of the Bonferroni-adjusted *p*-value (−log_10_), with larger values indicating higher statistical significance.

We further analyzed the raw data of miRNA profiles (MIRA) for these 100 patients among the three groups with varied time sequencing of miRNA and mRNA. Interestingly, out of the 89 miRNAs analyzed, 18 exhibited differential expression among the three groups (*p* < 0.05), while the remaining miRNAs showed consistent expression levels. A total of 15 of 18 miRNAs (miR-100-5p, miR-9-5p, miR-196b-5p, let-7c-5p, let-7d-5p, let-7g-5p, miR-126-3p, miR-222-3p, miR-152-3p, miR-145-5p, miR-143-3p, miR-181b-5p, miR-29a-3p, miR-30a-5p, and miR-26a-5p) are significantly different in the Slow groups compared to those in the Fast and Equal groups (Figure 1). After GO term analysis of these 15 miRNAs, we identified that aging is the most significant biological process that relates to these 15 miRNAs (Figure 2).

## 3. Discussion

The present study advances the understanding of endometrial receptivity by investigating the synchrony or asynchrony between microRNA (miRNA) and messenger RNA (mRNA)-based classification, exploring how these profiles contribute to successful embryo implantation (clinical pregnancy) in patients experiencing RIF and EBT. This approach is innovative in examining both the endometrial receptivity array (ERA) and microRNA receptivity assay (MIRA) in a single endometrial biopsy, providing a nuanced understanding of how the asynchrony of miRNA and mRNA expression impacts endometrial receptivity.

The definition of “repeated implantation failure (RIF)” is another key element for the present study. In the present study, we recruited those patients with RIF, according to the traditional criteria [20]. To avoid the confounding effect of aneuploidy embryos, all the patients participating in this study received at least one EBT failure prior to the present study. By this way, we could focus on the endometrial receptivity in the present study. Furthermore, we could achieve a high pregnancy rate in the Fast (94.1%) and Equal (84.9%) groups. A previous work in the literature highlighted that <5% of ART patients experience true RIF unlinked to embryonic aneuploidy [21]. Such high pregnancy rates in the Fast and Equal groups may reflect adequate endometrial receptivity and exclusion of aneuploidy. By contrast, the relatively low pregnancy rate (54.5%) in the Slow group may result from the truly downregulated endometrial receptivity. Nonetheless, a substantial abortion rate was noted in the Fast and Equal groups. Consequently, the live birth rates did not reach a statistically significant difference among the three groups. We may need 200 patients to detect a difference of 20% (76.5% vs. 54.5%) in live birth rates.

Our results reveal a significant association between the synchrony receptive stages between miRNA and mRNA profiles and clinical pregnancy (implantation) outcomes. The ‘Slow’ group refers to patients whose miRNA-based classification suggests a later receptive stage than the mRNA-based classification, indicating potential asynchrony in receptivity staging. The Slow group displayed notably lower pregnancy rates compared to the Fast and Equal groups. This finding underscores that the synchrony of receptive stages featured the pattern of miRNA following mRNA is crucial for creating an optimally receptive endometrial environment. Previous studies have focused largely on mRNA profiles alone [7,23], yet the emerging evidence here supports the inclusion of miRNA profiles to capture additional regulatory mechanisms [24,25]. MiRNAs are recognized for their post-transcriptional regulation of gene expression, modulating processes such as immune response, cell adhesion, and tissue remodeling, which are essential to embryo implantation [11,15].

The rate of 72% classification concordance based on receptivity staging between the ERA and MIRA suggests that while both tests provide valuable insights into endometrial receptivity, they measure varied aspects of gene expression and regulatory mechanisms. The moderate Kappa statistic (0.50) indicates that mRNA and miRNA profiles are complementary but not entirely overlapping in their assessment of receptivity. Given that miRNAs often target mRNAs for degradation or inhibition, the presence of miRNA may signify the preparation of endometrial tissue to modulate specific protein expressions in response to mRNA signals. Since the receptive stages of miRNA-based classification closely follow or overlap with the receptive stages of mRNA-based ones, maximizing the endometrial environment, this regulatory role of miRNAs may help to explain the higher pregnancy rates in the Fast and Equal groups.

The 18 miRNAs identified with differential expressions between the Fast, Equal, and Slow groups further highlight the complexity of miRNA involvement in endometrial receptivity. We conducted further analysis on the 15 predominant miRNAs, which significantly contributed to the distinct profile of the Slow group compared to the others.

Several of the mentioned miRNAs play important roles in endometrial function and female reproduction. First, the let-7 family (let-7c-5p, let-7d-5p, let-7g-5p) is associated with endometrial receptivity [24,26]. These miRNAs are likely to contribute to regulating gene expression during the implantation window. Second, miR-222-3p modifies the expression of CDKN1C/p57kip2, which is involved in endometrial stromal cell decidualization and differentiation [26]. Third, miR-145-5p is downregulated in the endometrium during the implantation window compared to the proliferative phase [26,27]. It is also reduced in the endometrium of pregnant mares, suggesting a role in embryo attachment, adhesion, and implantation [28]. Fourth, the miR-181 family regulates leukemia inhibitory factor (LIF) expression in the murine endometrium, which is important for endometrial receptivity and implantation [24]. Fifth, the miR-30 family (miR-29a-3p and miR-30a-5p) is upregulated in the receptive endometrium compared to the pre-receptive endometrium in healthy women [26,27]. These miRNAs have been reported to be relevant to endometrial receptivity. Furthermore, many miRNAs are involved in regulating endometrial function and may contribute to embryo–maternal dialogue [28].

These miRNAs play crucial roles in regulating gene expression related to endometrial receptivity, decidualization, and embryo implantation. They modulate various signaling pathways, including Wnt/β-catenin, ERK/MAPK, and TGF-β, which control transcription, cell proliferation, and apoptosis in the endometrium [29]. Furthermore, a bioinformatic overlay showed several miRNAs (e.g., let-7 family) targeting ERA panel genes (e.g., LIF), which indicates that miRNA may have a regulatory role on mRNA in the endometrium. Dysregulation of these miRNAs may contribute to fertility issues such as RIF [30].

According to functional study, these miRNAs are mainly linked to the mRNA related to aging signaling, suggesting that senescence-associated alterations in endometrial cells may be caused by or result from a lagging miRNA-based classification of receptive stage. Lower implantation rates in the Slow group may result from these alterations, which may impact the cellular environment’s preparedness for implantation. These results highlight the necessity of a more thorough diagnostic strategy that incorporates both the MIRA and ERA in order to better capture the intricate regulatory milieu required for endometrial receptivity. According to available data, there is still some clinical ambiguity when the MIRA receptive staging system and the ERA are not in synchrony. We suggested that the MIRA might offer further refinement, but we were unable to take into account the possibility that the MIRA could overturn ERA findings.

The asynchrony between miRNA- and mRNA-based receptive stagings observed more frequently in cases with endometrial polyp undergoing transcervical resectoscope suggests that the clinical endometrial factors could influence the miRNA–mRNA expression patterns, potentially modifying endometrial receptivity. This finding aligns with previous studies identifying endometrial polyps as a risk factor for implantation failure [16]. These insights point toward personalized strategies that consider such individual clinical factors when assessing the optimal window for embryo transfer.

While the findings of this study offer valuable insights into the synchrony of miRNA- and mRNA-based classification of stages in endometrial receptivity, several limitations should be considered. First, although the sample size is sufficient for initial exploration, it limits the generalizability of the results. A larger cohort would be necessary to confirm these findings across a broader population and to refine the receptive stage classification for clinical applications. Second, the study relied on two diagnostic assays (ERA and MIRA) which, while well-established, may not capture the full spectrum of molecular interactions that influence endometrial receptivity. We have the raw data for the MIRA but not for the ERA. Therefore, we were unable to confirm the gene enrichment analysis using the raw data from the ERA. Future studies incorporating next-generation sequencing or proteomic analyses could provide a more comprehensive view of the molecular landscape. Additionally, the impact of other potential confounding factors, such as lifestyle, genetic predispositions, and hormonal variations, was not fully explored, which could further influence the miRNA–mRNA interplay. Lastly, as the study focused on patients with RIF, the findings may not be directly applicable to other populations, such as women without a history of implantation issues, highlighting the need for diverse patient representation in subsequent research.

## 4. Materials and Methods

### 4.1. Study Design and Patient Population

This study was conducted to evaluate the concordance between two diagnostic tools, the microRNA receptivity assay (MIRA) and the endometrial receptivity array (ERA), in assessing endometrial receptivity in patients with recurrent implantation failure (RIF) undergoing euploidy blastocyst transfer (EBT). A total of 100 patients who had undergone repeated unsuccessful in vitro fertilization (IVF) attempts at either other hospitals or the Lee Women’s Hospital, Taichung, Taiwan was enrolled. Inclusion criteria included patients aged 20–49 years with a history of at least three previous failed embryo transfers or two previous failed embryo transfers including at least one euploid embryo transfer failure. Patients with endometrial abnormalities, such as fibroids or significant endometrial synechiae, were excluded from the study. Patients with untreated endometrial abnormalities were excluded; those with previously treated polyps or minor findings were included if normal hysteroscopy was confirmed before biopsy.

The study was conducted in accordance with the ethical standards of the institutional research committee and with the 1964 Helsinki declaration and its later amendments. Informed consent was obtained from all individual participants included in the study. The protocol was reviewed and approved by the local ethics committee of Chung Shan Medical University Hospital (CS2-22020) prior to the commencement of the study.

Endometrial tissue samples were obtained from each patient during the window of implantation (WOI), which was determined based on the patient’s menstrual cycle. The endometrium was prepared by hormone replacement for frozen embryo transfer. Biopsies were performed during the mid-luteal phase, typically at 116–124 h after the commence of progesterone supplement based on hormonal preparation protocols, minimizing intra-group timing differences. The timing for endometrial biopsy was selected according to the instructions from the manufacture of ERA tests. One biopsy was taken from each patient, part of the tissue for mRNA analysis (ERA) and another part for microRNA analysis (MIRA).

A total of 100 patients were included, each underwent a single endometrial biopsy divided for simultaneous ERA and MIRA analyses. Given the exploratory nature of this study, no formal sample size calculation was performed. However, a post hoc power analysis showed 80% power to detect a 30% difference in pregnancy rates between groups with alpha = 0.05. These patients underwent a personalized embryo-transfer protocol based on the results of the ERA. Trophectoderm biopsy was performed on day 5/6 blastocysts and PGT-A conducted via NGS. Only euploid embryos were transferred.

### 4.2. Endometrial Receptivity Array (ERA) Analysis

The ERA test was conducted to assess the mRNA expression profile associated with endometrial receptivity. RNA was extracted from the endometrial biopsy sample using a commercial RNA isolation kit. The RNA was then reverse-transcribed into cDNA and analyzed using a custom gene expression microarray specifically for endometrial receptivity. The gene expression profiles were classified into three categories: pre-receptive (P + 4), receptive (P + 5), and post-receptive (P + 6). The classification of pre-receptive, receptive, and post-receptive was performed by the service provider (Igenomix, Taipei, Taiwan). Results were used to determine the timing of the window of implantation for each patient.

The ERA is composed of 238 mRNAs, which are listed in the published literature [7], Nonetheless, due to proprietary restrictions, raw ERA gene expression data were not available; only classification output was provided by the service provider.

### 4.3. MicroRNA Receptivity Assay (MIRA) Analysis

For the MIRA test, the biopsied endometrial tissue was used to extract microRNAs. Total RNA was isolated from up to 30 μg of endometrial tissue with miRNeasy Tissue/Cells Advanced mini Kit (Cat. No. 217604, Qiagen, Taipei, Taiwan) following the manufacturer’s protocol. Total RNA samples were eluted in 60 μL nuclease-free water. The concentrations of the extracted total RNAs were quantified using Qubit RNA HS Assay Kit (Q32852, Thermo-Fisher, Waltham, MA, USA).

A total of 25 ng of total RNA samples were used to synthesize cDNA in 20 μL reverse transcription reactions. The reverse transcription step was performed as follows: Poly-A tail was added to the RNA population using Poly-A polymerase, followed by cDNA synthesis with RNA Reverse Transcription kit (Quark Biotechnology, Hsinchu, Taiwan). qPCR was performed utilizing NextAmp™ Analysis System (Quark Biotechnology) and MIRA^TM^ miRNA-based Endometrial Receptivity Analysis PanelChip^®^ (Inti Labs, Hsinchu, Taiwan) with preprinted microRNA specific primers. For qPCR analysis, 2.5 ng cDNA was added to the qPCR master mix (Quark Biosciences, Inc., Hsinchu, Taiwan), and qPCR was performed on Q Station (Quark Biosciences, Inc.) according to the following program: 95 °C for 36 s and 60 °C for 72 s for 40 cycles.

The miRNA expression was profiled using a customized miRNA panel, consisting of 89 miRNAs associated with endometrial receptivity. The experiment on MIRA™ PanelChip^®^ generated 89 miRNAs’ expression profiles data. A proprietary algorithm for MIRA™ (Inti Labs) processed the miRNAs’ expression profiles to identify the window of implantation (WOI). Similar to the ERA, the results were categorized into pre-receptive, receptive, and post-receptive phases based on the miRNA expression patterns by the service provider (Inti Labs).

### 4.4. Group Classification Based on miRNA and mRNA Timing

Patients were further classified into three groups based on the synchrony or asynchrony of receptive stages between miRNA and mRNA profiles: Fast (miRNA-based classification stages preceded mRNA-based classification ones), Equal (miRNA- and mRNA-based classification stages are concordant), and Slow (miRNA-based classification stages lagged behind mRNA-based classification ones). Namely, the ‘Slow’ group refers to patients whose miRNA-based classification suggests a later receptive stage than the mRNA-based classification. This grouping category was used to explore the impact of asynchrony of receptive stages according to miRNA and mRNA profiles on pregnancy outcomes.

### 4.5. Personalized Embryo Transfer (ET) and Pregnancy Outcomes

Following the analysis, personalized embryo transfers (ET) were scheduled for each patient based on their ERA results. Frozen embryo transfers (FET) with euploidy blastocysts were performed in subsequent cycles at the time designated as receptive according to the ERA results. Pregnancy outcomes were then monitored and recorded, with success defined as a viable pregnancy confirmed by ultrasound.

### 4.6. Statistical Analysis

To assess the synchrony between the ERA and MIRA results, each patient’s mRNA- and miRNA-based receptivity stages were compared. A Kappa statistic was calculated to measure the level of agreement between the ERA and MIRA tests. The concordance was determined by categorizing the results into three groups within two categories: concordant (ERA and MIRA results matched; the Equal group), and discordant (asynchrony results between the two tests; the Fast and Slow groups).

Further statistical analyses were performed using standard methods. The chi-square test (X^2^) was used to compare categorical variables, and Kruskal–Wallis test was used for continuous variables. Clinical pregnancy rates across the Fast, Equal, and Slow groups were compared using chi-square tests, and *p*-values less than 0.05 were considered statistically significant. All analyses were conducted using statistical software. MedCalc Statistical Software version 19.1 (MedCalc Software bv, Ostend, Belgium).

### 4.7. Functional Enrichment Analysis

We used TAM2.0 [31] to perform a functional enrichment analysis of miRNAs. We set the screening criteria so that the size of the miRNA category has at least two miRNAs, each functional enrichment result must include at least two miRNAs, and we apply the Bonferroni correction with a *p*-value threshold of <0.01.

## 5. Conclusions

The synchrony or asynchrony of receptive stages between miRNA and mRNA classification are intimately associated with pregnancy outcomes in patients with RIF and EBT. The findings emphasize that miRNA profiles, when assessed alongside traditional mRNA-based diagnostics, provide additional insights into the regulatory dynamics of the endometrium, meaning they might enhance the precision of personalized embryo transfer protocols.

The observed asynchrony of receptive stages in miRNA and mRNA profiles, associated with factors such as endometrial polyp, underscores the need for a multifactorial approach to understanding endometrial receptivity. Future studies should continue to explore the mechanistic pathways linking miRNA and mRNA expression with endometrial receptivity, focusing on developing more sophisticated diagnostic tools that integrate miRNA data. Ultimately, a dual-assessment approach could offer a more holistic and tailored method for managing implantation challenges in IVF protocols.

## Figures and Tables

**Table 1:** The results of MIRA (microRNA profiles) and ERA (mRNA profiles) for endometrial receptivity assay in patients with recurrent implantation failure. We categorized the results into pre-receptive (P + 4), receptive (P + 5), and post-receptive (P + 6) groups based on their ERA or MIRA tests. P denotes the onset of progesterone effect.

ERA (mRNA Profiles)	MIRA (microRNA Profiles)			Total
	Pre-Receptive (P + 4)mi	Receptive (P + 5)mi	Post-Receptive (P + 6)mi	
Pre-receptive (P + 4)m	13	9	0	22 (22.0%)
Receptive (P + 5)m	6	51	8	65 (65.0%)
Post-receptive (P + 6)m	1	4	8	13 (13.0%)
Total	20 (20.0%)	64 (64.0%)	16 (16.0%)	100

Kappa statistic for concordance between these two tests is 0.50 (95% CI: 0.34–0.66).

**Table 2:** Pregnancy outcomes after personalized embryo transfer according to ERA results; the patients are further divided into three groups by the dating time sequence between miRNA [(P + i)mi] and mRNA [(P + j)m] profiles. We grouped the patients into Fast [(P + i)mi vs. (P + j)m, i < j], Equal [(P + i)mi vs. (P + j)m, i = j], and Slow [(P + i)mi vs. (P + j)m, i > j] categories (i or j = 4 or 5 or 6).

Pregnancy Rate	Fast (n = 17)	Equal (n = 72)	Slow (n = 11)	*p* ^1^
ERA pre-receptive	8/9 (88.9%)	10/13 (76.9%)	NA	0.485
ERA receptive	8/8 (100%)	42/51 (82.4%)	4/6 (66.7%)	0.247
ERA post-receptive	NA	7/8 (87.5%)	2/5 (40.0%)	0.083
Total	16/17 (94.1%)	59/72 (81.9%)	6/11 (54.5%)	0.031

^1^ *p* value by X^2^ tests, NA denotes not available.

**Table 3:** The association of clinical characteristics in the miRNA (MIRA) and mRNA (ERA) profiles for endometrial receptivity, grouping by the dating staging sequence between miRNA [(P + i)mi] and mRNA [(P + j)m] profiles. We grouped the patients into Fast [(P + i)mi vs. (P + j)m, i < j], Equal [(P + i)mi vs. (P + j)m, i = j], and Slow [(P + i)mi vs. (P + j)m, i > j] categories (i or j = 4 or 5 or 6). The data are presented with median (interquartile range) or number (percentage). BMI, AMH, and PCOS denote body mass index, anti-Mullerian hormone, and polycystic ovarian syndrome, respectively.

Clinical Factors	Fast (n = 17)	Equal (n = 72)	Slow (n = 11)	*p* ^1^
Age (Y)	36.0 (34.0–42.0)	39.0 (36.5–43.0)	40.0 (35.5–45.5)	0.260
BMI (Kg/m^2^)	20.7 (18.8–23.1)	21.5 (20.3–23.4)	21.1 (19.3–23.1)	0.524
Duration of infertility (Y)	2.4 (1.7–4.1)	3.3 (2.0–5.2)	4.0 (1.5–6.8)	0.633
AMH (ng/mL)	1.52 (0.22–3.21)	2.07 (0.70–3.91)	2.09 (0.88–7.03)	0.334
Embryo transfer times	3.0 (2.0–3.3)	3.0 (2.0–3.0)	3.0 (2.0–3.8)	0.875
PCOS (%)	2 (11.8%)	9 (12.5%)	4 (36.4%)	0.109
Endometriosis (%)	5 (29.4%)	17 (23.6%)	3 (27.3%)	0.869
Endometrial polyps (%)	10 (58.8%)	23 (31.9%)	2 (18.2%)	0.019
Clinical pregnancy (%)	16 (94.1%)	59 (81.9%)	6 (54.5%)	0.013
Abortion (%)	3 (17.6%)	7 (9.7%)	0 (0%)	0.128
Live birth (%)	13 (76.5%)	52 (72.2%)	6 (54.5%)	0.251

^1^ *p* value by Kruskal–Wallis test or X^2^ for trend tests.

## Data Availability

The raw qPCR-based microRNA array data generated in this study, encompassing expression profiles from 100 individuals, are available upon reasonable request. The dataset includes Ct values for 96 probes per sample, comprising both target microRNAs and internal control probes, including miRNA extraction control, miRNA reverse-transcription (RT) control, and qPCR amplification control. Due to ethical considerations and participant privacy, de-identified data will be shared for research purposes only, subject to Institutional Review Board (IRB) approval, where applicable.
